# Antifreeze Peptides and Glycopeptides, and Their Derivatives: Potential Uses in Biotechnology

**DOI:** 10.3390/md11062013

**Published:** 2013-06-10

**Authors:** Jeong Kyu Bang, Jun Hyuck Lee, Ravichandran N. Murugan, Sung Gu Lee, Hackwon Do, Hye Yeon Koh, Hye-Eun Shim, Hyun-Cheol Kim, Hak Jun Kim

**Affiliations:** 1 Division of Magnetic Resonance, Korea Basic Scienc Institute, Chungbuk 363-833, Korea; E-Mails: bangjk@kbsi.re.kr (J.K.B.); ravi@kbsi.re.kr (R.N.M.); 2 Division of Polar Life Sciences, Korea Polar Research Institute, Incheon 406-840, Korea; E-Mails: junhyucklee@kopri.re.kr (J.H.L.); holynine@kopri.re.kr (S.G.L.); kordhw@kopri.re.kr (H.D.); hykoh@kopri.re.kr (H.Y.K.); wow3300@kopri.re.kr (H.-E.S.); 3 Department of Polar Sciences, University of Science and Technology, Incheon 406-840, Korea; 4 Division of Polar Climate Research, Korea Polar Research Institute, Incheon 406-840, Korea; E-Mail: kimhc@kopri.re.kr

**Keywords:** antifreeze glycopeptide, antifreeze protein, recrystallization inhibition, thermal hysteresis

## Abstract

Antifreeze proteins (AFPs) and glycoproteins (AFGPs), collectively called AF(G)Ps, constitute a diverse class of proteins found in various Arctic and Antarctic fish, as well as in amphibians, plants, and insects. These compounds possess the ability to inhibit the formation of ice and are therefore essential to the survival of many marine teleost fishes that routinely encounter sub-zero temperatures. Owing to this property, AF(G)Ps have potential applications in many areas such as storage of cells or tissues at low temperature, ice slurries for refrigeration systems, and food storage. In contrast to AFGPs, which are composed of repeated tripeptide units (Ala-Ala-Thr)*_n_* with minor sequence variations, AFPs possess very different primary, secondary, and tertiary structures. The isolation and purification of AFGPs is laborious, costly, and often results in mixtures, making characterization difficult. Recent structural investigations into the mechanism by which linear and cyclic AFGPs inhibit ice crystallization have led to significant progress toward the synthesis and assessment of several synthetic mimics of AFGPs. This review article will summarize synthetic AFGP mimics as well as current challenges in designing compounds capable of mimicking AFGPs. It will also cover our recent efforts in exploring whether peptoid mimics can serve as structural and functional mimics of native AFGPs.

## 1. Introduction

In order to survive in extremely cold environments, many organisms have evolved unique adaptive mechanisms. Nearly two-thirds of the surface of the earth consists of water, with the average surface temperature of seas and oceans varying from −2 °C to 30 °C depending on the latitude. Within the polar regions, seawater temperatures are consistently subzero because of the presence of dissolved salts. These subzero temperatures can be extremely harmful, if not deadly, to polar fish. As a cold-adaptation mechanism, various polar fish produce antifreeze proteins/peptides (AFPs) or antifreeze glycopeptides (AFGPs) to protect themselves from freezing damage. These proteins and peptides have ice-binding affinity, and can lower the freezing temperature of a solution non-colligatively without affecting its melting temperature, a phenomenon termed thermal hysteresis (TH). In addition, AF(G)Ps can inhibit ice recrystallization, in which large ice crystals grow at the expense of smaller ones, and thus prevent mechanical cell damage caused by ice regrowth. Currently, it is generally accepted that AF(G)Ps function through the adsorption of their flat ice-binding surfaces onto particular planes of ice crystals, and thus prevent or inhibit further ice growth [1,2]. AF(G)Ps are produced by diverse fish species and are classified into 5 distinct categories (types I–IV and AFGP) depending on their structural characteristics and source [3,4]. AF(G)Ps are remarkable examples of convergent evolution because they have entirely different protein sequences and structures but perform the same function. Elucidation of the critical features responsible for the interaction of AF(G)Ps with ice crystals has been complicated by the wide range of molecular weights, sequences, and structures of AF(G)Ps. Experimental procedures that have been used to decipher the molecular mechanism include ice-etching studies [5], site-directed mutation studies [6,7,8,9,10,11,12], and structural studies, including nuclear magnetic resonance (NMR) spectroscopy [13,14,15], Fourier transform infrared spectroscopy [16], and X-ray [17,18]. In recent years, several AFPs and ice-binding proteins (IBPs) have also been identified and characterized from insects [19,20,21,22], fungi [23,24,25], yeast [26,27,28], bacteria [29,30,31], and plants [32,33]. Notably, insect AFPs have much stronger antifreeze activity than fish Af(G)Ps with a specific ice-binding motif, such as T-X-T or T-X-N. The hyperactive AFPs from *Choristoneura fumiferana* [19] and *Tenebrio molitor* [22] have a β-helical structure, whereas the hyperactive AFP from the snow flea has a bundle of six tightly packed polyproline type II helical coils and a hydrophobic ice-binding face without threonine residues [34,35].

Among the several types of AFPs, Type I AFPs were the first reported and have been extensively studied. Type I AFPs are alanine-rich peptides found in blood samples from winter flounder (*Pseudopleuronectes americanus*) and shorthorn sculpin (*Myoxocephalus scorpius*). To date, three-dimensional (3D) structures of the AFPs HPLC6 and ss3 have been solved [36,37] and their ice-binding residues have been identified using structure-based mutagenesis studies. Accumulated structure-function relationship studies on Type I AFPs have revealed that common Ala-rich hydrophobic regions are essential for potent antifreeze activity [36].

The first AFGP gene was characterized from the Antarctic notothenioid (*Notothenia coriiceps*) and its gene products possess Thr-*O*-linked glycosides. A typical AFGP consists of repeating tripeptide units, the alanyl-alanyl-threonyl (Ala-Ala-Thr)*_n_*_=4–50_ unit, connected to the disaccharide β-d-galactosyl-(1→3)-α-d-*N*-acetylgalactosamine through a glycosidic bond at the second hydroxyl group of the threonine residue. Eight distinct AFGP subtypes exist in nature. Among them, AFGP1 has the largest weight (33.7 kDa), and AFGP8 the lowest (2.6 kDa). In contrast to antifreeze glycoproteins, glycoproteins and glycopeptides are well known and extensively studied as biomolecules that play important roles in various biological processes such as immune differentiation, cell adhesion, cell differentiation, and cell growth regulation. Surprisingly, a tumor-associated carbohydrate antigen (T-antigen) found in mammals also contains the same glycosyl residue [38].

AF(G)Ps are believed to directly interact with the ice surface, resulting in freezing point depression to a level more than 200- to 300-fold greater than ordinary cryoprotectants (sugars and polyols) or salts, on a molar basis [39]. The ability of AF(G)Ps to influence ice crystal growth has led to a wide variety of applications such as improving the properties of frozen foods, cryopreservation of transplant organs and cells, cryosurgery, and aquaculture [4,40,41]. In contrast to AFPs that are accessible by recombinant protein expression techniques, AFGPs can be obtained only in small amounts as pure samples by chemical synthesis. Chemical and biochemical approaches for obtaining homogeneous AFGP are much more demanding because of the presence of the anomeric carbon-oxygen bond in native AFGPs. Moreover, other factors that must be overcome before AF(G)Ps can be used in widespread applications are cytotoxicity and cellular damage driven by their dynamic ice-shaping ability [5,6,42,43,44]. This review focuses on the marine type I family of AFPs and AFGPs. More detailed information about other types of AFPs and IBPs is available in other review articles [45,46,47].

## 2. Antifreeze Activity Assays

In the antifreeze activity assay of AF(G)Ps, two experimental techniques have been used, *i.e.*, thermal hysteresis (TH) assay [48] and ice recrystallization inhibition (RI) assay [49]. In the TH assay, a temperature gap between freezing and melting point of a solution in the presence of AF(G)Ps is measured by using nanoliter osmometer instrument connected with a microscope to examine the ice morphology and growth. Since the freezing and melting points are determined by direct observation of scientist, this assay involves the time-consuming and laborious task. In order to avoid this inconvenience, a method using differential scanning calorimeter (DSC) was proposed to measure the thermal hysteresis activity of AF(G)Ps [50]. DSC provides more accurate and repeatable TH value of AF(G)Ps without the need of microscopic observation but the high cost of DSC instrument is the disadvantage of this method.

The RI assay measures the size of ice crystals in AF(G)P solution using a microscope at frozen temperature. If AF(G)P has strong RI activity, smaller ice crystals are visible but control sample without AF(G)P shows large ice crystals during the process. This method is much simpler and faster than TH assay. However, it is more suitable to check the antifreeze activity is present or not because it is very difficult to obtain quantitative results.

## 3. Type I Antifreeze Peptides

**Figure 1:**
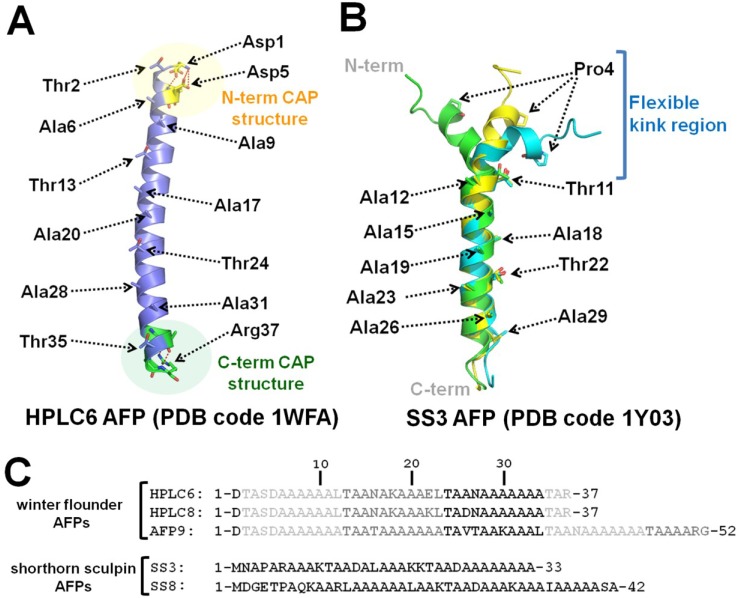
Representative structures of Type I antifreeze proteins (AFPs). (**A**) Overall structure of HPLC6 Type I AFP (PDB code 1WFA) from winter flounder. Thr- and Ala-rich ice-binding residues are labeled. *N*- and *C*-terminal cap structures are also shown in yellow and green, respectively. (**B**) Three different conformers of the ss3 AFP (PDB code 1Y03) solution structure from shorthorn sculpin are shown in cartoon representation. Thr and Ala residues constituting the ice-binding sites are labeled and the *N*-terminal flexible kink region is also indicated. (**C**) Amino acid sequences of representative Type I AFPs.

Type I AFPs have single α-helices and are found in winter flounder (*Pseudopleuronectes americanus*) and shorthorn sculpin (*Myoxocephalus scorpius*). The most extensively characterized type I AFP is the winter flounder HPLC6 AFP, which has 37 residues and high alanine content (23 residues are alanine). It has three repeated sequences, which contain 11 amino acid repeats; Thr-Ala-Ala-X-Ala-X-X-Ala-Ala-X-X, where X can be any amino acid. The crystal structure of the HPLC6 AFP (PDB code 1WFA) [36] revealed that this has an amphipathic right-handed α-helical conformation with *N*- and *C*-terminal cap structures to ensure its stability in solution. The *N*- and *C*-terminal cap structures are stabilized by a hydrogen bond network that involves structurally ordered water molecules, and help the protein maintain its helicity (Figure 1A). Previous site-directed mutagenesis and docking simulation studies proposed that the Ala-rich hydrophobic face of HPLC6 AFP is the primary ice-binding site [51].

Shorthorn sculpin ss3 AFP is another Type I AFP, which also has high alanine content (21 alanine residues out of a total of 33 residues) [37]. Unlike HPLC6 AFP, ss3 AFP contains a proline residue, which is a helix-breaking amino acid, at position 4. The structure of the shorthorn sculpin AFP isoform ss3 (PDB code 1Y03) [52] was determined by NMR spectroscopy. The overall structure of ss3 AFP is an α-helical peptide, similar to HPLC6 AFP, with residues 7–11 appearing to form a flexible kink region because of Pro4 (Figure 1B).

AFP9 from winter flounder is a longer type I AFP compared with HPLC6 AFP and has 4 repeat sequences [53]. The TH activity of AFP9 (1.0 °C at 10 mg/mL) is much higher than that of HPLC6 AFP (0.68 °C at 10 mg/mL), illustrating the general effect of increased size on TH activity. More recently, a much larger type I AFP (195-residue protein) was discovered in winter flounder (*Pseudopleuronectes americanus*) with a molecular weight of 17 kDa. This protein is five times larger than HPLC6 AFP, and it exhibits significantly high TH activity even at low concentrations. Hence, this protein was named as hyperactive type I AFP [54]. Although a high-resolution structure of hyperactive type I AFP has not yet been determined, circular dichroism data and analytical ultracentrifugation results suggest that the protein is a side-by-side dimer of two α-helices [55].

## 4. Structure-Activity Relationship (SAR) Studies of Type I AFP

To identify key residues for ice binding, structure-activity relationship studies were performed on HPLC6 AFP using truncated or synthetic analogs. To date, over 50 mutant peptides of HPLC6 AFP have been produced and their antifreeze activities have been compared to that of wild-type HPLC6 AFP. It was initially suggested that Thr residues are critical for hydrogen bond interactions with water molecules on the ice surface. To test this hypothesis, Thr residues in HPLC6 AFP were replaced with Ser (T13S and T24S double mutant), which was expected to maintain the hydrogen bond with ice through its hydroxyl group. Unexpectedly, the mutation significantly reduced the TH activity. On the other hand, Thr to Val mutants (T13V and T24V double mutant) retained most of the TH activity (85% TH activity compared to that of wild-type HPLC6 AFP), indicating that the hydrophobic methyl group of Thr is important for ice binding in HPLC6 AFP [7]. Therefore, subsequent mutational studies focused on the conserved hydrophobic Ala residues just next to the Thr residues in HPLC6 AFP. The A17L mutant resulted in a complete loss of TH activity and the A21L mutant showed 10% TH activity compared with wild-type HPLC6 AFP, suggesting that the Ala17 and Ala21 residues are located in the ice-binding region and that the bulky side chain of Leu sterically inhibited HPLC6 AFP binding to ice [12]. Taken together, the Ala and Thr methyl groups participate in the formation of a hydrophobic ice-binding surface, and these residues have been redefined as the true ice-binding site of HPLC6 AFP. In a series of articles, further mutational studies demonstrated that the salt bridge between Lys18 and Glu22, which helps stabilize the helix, is not essential for activity [11]. Truncated HPLC6 AFP was constructed to identify the minimal sequence having antifreeze activity: truncated analogues of *N* and/or *C*-terminal residues were chemically synthesized, and their antifreeze activities examined. Furthermore, to investigate the relationship between the α-helicity and antifreeze activity of the peptides, the circular dichroism (CD) spectra of the peptides were analyzed. The decreased α-helical content of these peptides directly correlated with the decrease in their antifreeze activity. These results showed that the α-helical conformation is essential for the antifreeze activity of Type I AFP. To improve solid-phase synthesis and aqueous solubility, Haymet *et al*. have incorporated 2 salt bridges (A7K/A11E and A29K/A33E) into HPLC6 AFP. The mutants retained similar TH and recrystallization inhibition (RI) activities compared with wild-type HPLC6 AFP, but their solubilities were improved by the salt bridges [8]. In addition, the ice-binding residues and ice-binding mode of HPLC6 AFP were defined through a number of molecular modeling and simulation studies. Computational simulation studies also clearly confirmed that the Ala-rich region is a key element required for the ice binding of HPLC6 AFP.

Unlike HPLC6 AFP, shorthorn sculpin AFPs contain no repeat spacing of Thr; however, shorthorn sculpin AFPs have several Lys and Arg residues (K9, R12, K23, and K31 in ss8). Initial modeling studies showed that the positively charged residues can be matched with the ice crystal lattice, indicating the participation of the charged residues in ice binding of shorthorn sculpin AFPs [56]. For the shorthorn sculpin protein, the NMR solution structure of ss3 is available, but mutational analysis has been performed only on the ss8 isoform, and not on ss3 [57]. Nevertheless, mutagenesis data on ss8 can be used to suggest an ice-binding site for ss3 because of the high degree of sequence homology (over 80%) between ss3 and ss8. First, several Ala to Lys substitutions were introduced to investigate the effect of the mutation on the antifreeze activity of ss8 [57]. The A16K, A19K, and A22K mutations did not affect antifreeze activity, which remained almost the same as that of the wild type; however, single mutations (A17K, A21K, and A25K) on the hydrophobic surface of ss8 eliminated most of the antifreeze activity. Currently, it is generally accepted that the ice-binding site of the sculpin type I AFP is on the Ala-rich face of ss8 (Ala17, Ala21, and Ala25) similar to that seen in HPLC6 AFP [57]. Based on sequence alignment, the residues in ss3 (Ala12, Ala17, and Ala23) were found to be equivalent to the 3 ss8 residues that resulted in decreased TH activity when mutated. Interestingly, these residues all form a cluster on one side of the ss3 structure, suggesting that this site is the putative ice-binding face of ss3 (Figure 1C).

The ice-binding residues in hyperactive type I AFP have not yet been identified by mutation analysis, but sequence analysis and modeling studies have provided a possible ice-binding mechanism for hyperactive type I AFP [54]. The structural model of hyperactive type I AFP adopts a long α-helical conformation and forms an antiparallel homodimer with multiple Ala- and Thr-rich patches, similar to the ice-binding sites of HPLC6 and ss3 AFP. In other words, hyperactive type I AFP contains several ice-binding sites in a manner that increases its ice-binding affinity and antifreeze activity. HPLC6 AFP and ss3 AFP preferentially bind to the pyramidal plane and secondary prism plane of the ice lattice, respectively, but the ice plane interacting with hyperactive type I AFP has not yet been determined [5]. Nevertheless, the lemon shape and bursting growth of ice crystals in the presence of hyperactive type I AFP suggest that this protein can bind to more than one plane of the ice crystal. Therefore, it is thought that the hyperactivity stems from its multiple ice-binding activity and increased coverage of the ice surface [54].

In conclusion, the various type I AFPs have a common α-helical structure and many Ala residues, which constitute an Ala-rich hydrophobic ice-binding face. On the other hydrophilic side of the helix, charged or polar residues are involved in enhancing the solubility of Type I AFP.

## 5. Native Antifreeze Glycoproteins: Synthesis and Structure-Activity Relationship (SAR) Studies

**Figure 2:**
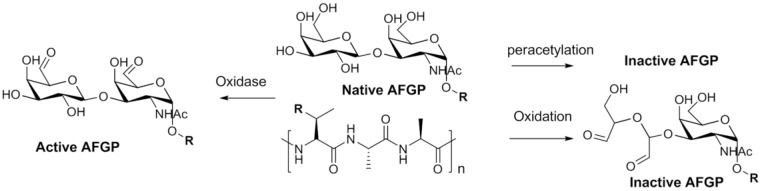
Key degradation studies on native antifreeze glycoprotein (AFGP).

The unique physiochemical properties of AFGPs have led to considerable interest in these molecules for various applications; however, the lack of progress in their mechanistic and SAR studies is largely because of the presence of numerous isoforms of AFGP in blood plasma and the difficulty in isolating them in pure form [58]. Moreover, the expression and purification of AFGPs by using molecular biotechnology techniques has not been successful thus far; therefore, chemical synthesis may be the only way to produce significant quantities of pure AFGP for detailed SAR studies. Without isolating and purifying the derivatives, early structure-property relationship studies on the sugars and peptide backbone were carried out using the standard carbohydrate and Edman degradation methods. These studies revealed that oxidation of the hydroxyl groups (80%) totally eliminated activity, except for the C-6 hydroxyl group, which tolerates a range of substituents with the exception of charged groups (Figure 2). With respect to peptide backbone modifications, the occasional substitution of Arg for Thr or Pro for Ala does not have a significant effect on non-colligative freezing point depression [59,60,61,62,63,64]. These studies highlight the importance of the sugar structure, especially the hydroxyl groups except for C-6, for significant antifreeze activity. Studies on the importance of structural elements have also been performed, such as the number of hydroxyl groups, stereochemistry of the sugar hydroxyls, acetamido group on the first galactose sugar, stereochemistry of the β-glycosidic linkage between the 2 sugars, α-glycosidic linkage to Thr, and requirement of disaccharide for activity. However, no detailed information was established until the first total synthesis and complete structure-activity relationship study on AFGP was undertaken by Nishimura and his co-workers [65,66,67,68].

**Figure 3:**
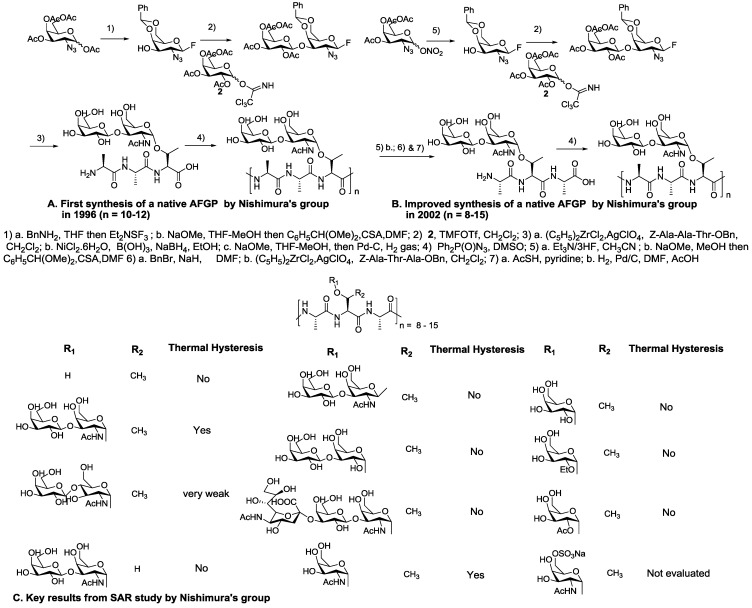
Synthesis and structure-activity relationships of native AFGP. Note: modified from [65,66,68].

They reported the first synthesis of naturally occurring AFGPs as a mixture of polymers with an estimated molecular mass of 6000–7300 Da (*i.e.*, *n* = 10–12), but did not report any of their TH, ice growth habit modification, or recrystallization inhibition properties. These synthetic antifreeze glycoproteins (syAFGP) were prepared by directly polymerizing an unprotected glycopeptide macromonomer in the presence of diphenylphosphorylazide (DPPA) as a promoter (Figure 3A). They synthesized the glycopeptide macromonomer from commercially available 1,3,4-tetra-*O*-acetyl-2-azido-2-deoxy-α/β-d-galactopyranose in 6 steps [65]. The same group further elaborated on this synthetic strategy, in which the amino acid sequence of the building block was modified from Ala-Ala-Thr to Ala-Thr-Ala, and the acetyl protecting groups were converted into benzyl protecting groups in order to reduce the steric hindrance of sugars during the activation of the terminal carboxyl groups and to prevent the racemization of β-elimination reactions due to *O*-deacylation of the intermediates under alkaline conditions (Figure 3B). Significant antifreeze activity (TH) was observed using syAFGPs containing 8–15 repeating units [66]. Subsequently, a practical method was recently reported for the preparation of sequential glycoproteins by replacing DPPA with 1-isobutoxycarbonyl-2-isobutoxy-1,2-dihydroquinoline (IIDQ) or 4-(4,6-dimethoxy-1,3,5-triazin-2-yl)-4-methylmorpholinium chloride (DMT-MM) with yields close to 90%, whereas DPPA gave complex mixtures of acyl migrated compounds [67]. Nishimura and his co-workers [68] in 2004 reported critical structure-activity data by using the same solution-phase polymerization method (Figure 3C). Numerous AFGP analogues were synthesized by modifying both the structure of the sugar moieties and the peptide backbone, and the antifreeze activity was observed. This summarized the essential framework of AFGPs that display significant antifreeze activity: the disaccharide must be α-configured to every Thr, the *N*-acetyl group has to be present at the C2 position, sugars must be *galacto*-configured, and the α-methyl group of the threonyl residue is critically required for potent inhibitory activity [68].

**Figure 4:**
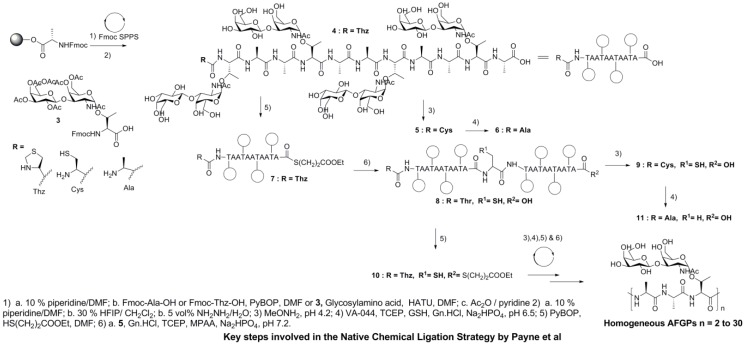
Synthesis of native AFGP analogues using native chemical ligation. Note: modified from [70].

However, the major drawback of this synthetic approach is that the polymerization strategy requires extensive HPLC purification to obtain pure compounds, which is not suitable to scaling up AGFP production. As a result, the first example of AFGP analogue synthesis used native chemical ligation, followed by the use of the Cys desulfurization method. Two consecutive chemical ligations of glycopeptides, each comprising 6 residues, followed by 3 more steps, yielded good amounts of the 21-residue AFGP, but these analogues have not been tested for their TH, ice growth habit modification, or recrystallization inhibition activities [69]. Recently, however, the same strategy has been applied to prepare homogeneous syAFGPs ranging in size from 1.2 to 19.5 kDa, which represents one of the largest native glycoproteins prepared by total chemical synthesis (Figure 4).

Specifically, the effectiveness of this strategy relies on 3 key reactions: (1) native chemical ligation between a glycopeptide bearing an *N*-terminal thiazolidine (Thz) residue and a *C*-terminal thioester moiety, and a glycopeptide containing an *N*-terminal cysteine (Cys) residue, (2) conversion of the *N*-terminal Thz moiety to an *N*-terminal Cys residue, and (3) desulfurization of the Cys-containing glycopeptides to afford native and homogeneous AFGPs [70].

## 6. Synthesis of AFGP Analogues

### 6.1. Modification of Sugars and Peptide Backbone

**Figure 5:**
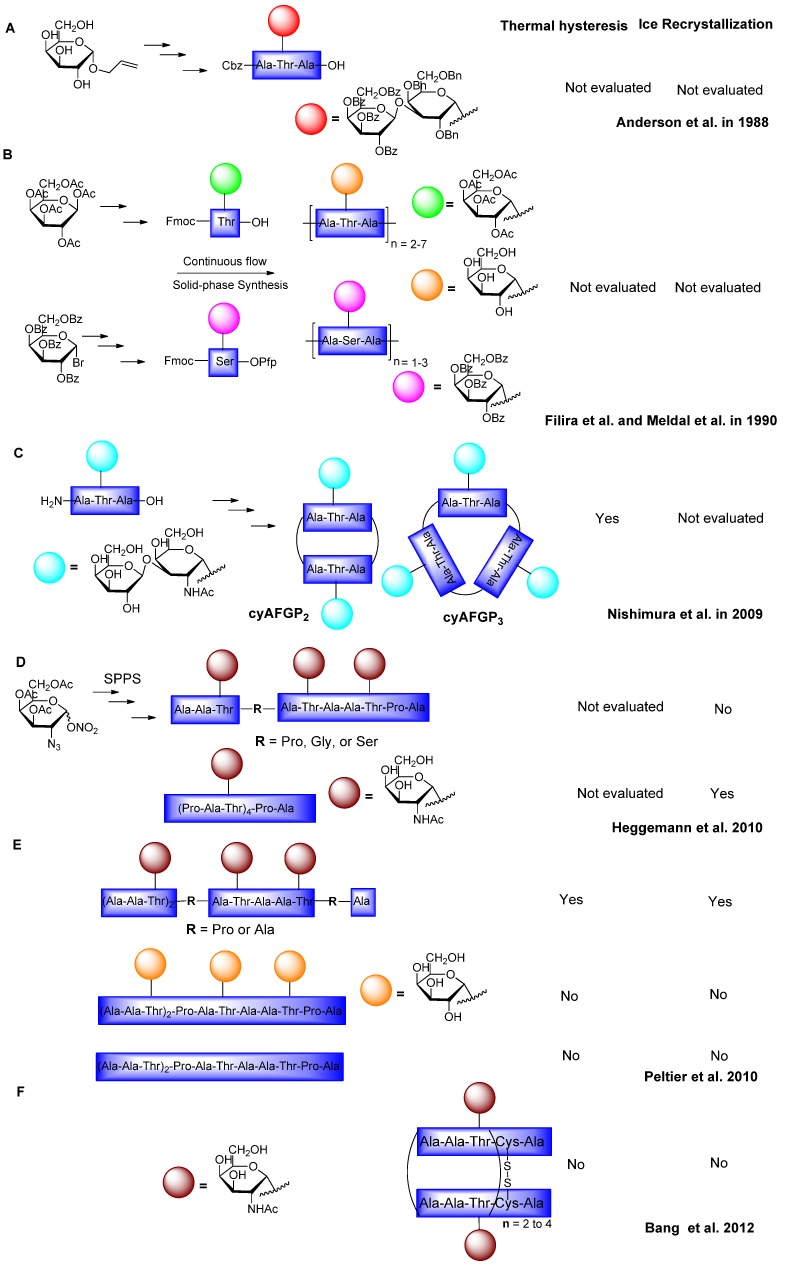
Synthesis of AFGP analogues and their antifreeze activities. Note: the figure is modified from [71,72,73,74,75,76].

The intrinsic factors that affect commercialization of native AFGP for medical and industrial applications are limited natural abundance and inherent instability of the C–O glycosidic bond. In an effort to address these issues, various research groups are involved in exploring whether AFGP analogues could serve as structural and functional mimics of native AFGP. The first synthesis of an AFGP analogue in which the β-d-galactosyl-(1,3)-α-d-*N*-acetylgalactosamine was substituted by β-d-galactosyl-(1,3)-α-d-galactose was reported in 1988 by Anderson and coworkers (Figure 5A). It involved the silver triflate-mediated direct glycosylation of the peptide backbone in the last step using disaccharide, in which the disaccharide and the protected tripeptide fragments were synthesized from the corresponding unprotected galactoside in five steps using typical peptide sequential coupling. However, the final glycosylated tripeptide was neither used to synthesize repeating AFGP analogues, nor did it possess antifreeze activity [71].

Related solid phase synthesis procedures for AFGPs containing single sugars were reported by Filira *et al.* and Meldal and Jensen [72,77] by employing continuous flow solid-phase techniques or a stepwise elongation of the peptide backbone using a glycoconjugate (Figure 5B). The advantage of solid-phase synthesis over the solution phase route is the ability to synthesis diverse AFGP analogues by varying the length, sequence, nature of amino acid residues, and carbohydrate moieties using the Fmoc-protected building block. In addition, this route avoids the tedious purification of a mixture of oligomers and does not require a single tripeptide unit for the polymerization reaction [72,77]. The report by Filira *et al*. [72] was the first attempt to demonstrate the utility of solid-phase synthesis for the preparation of range of glycopeptides with lengths varying between 6 and 21 residues using *N*-α-(fluoren-9-ylmethoxycarbonyl)-3-*O*-(2,3,4,6-tetra-*O*-acetyl-α-d-galactopyranosyl)-l-threonine as a symmetrical anhydride [72]. Similarly, Meldal *et al.* [77] synthesized AFGP analogues lacking the terminal galactose and the *N*-acetyl group, and with a substitution of the threonine residue in the alanyl-threonyl-alanyl (Ala-Thr-Ala) polypeptide with serine. Moreover, an interesting feature is the use of pentafluorophenyl ester (Pfp) to protect the *C*-terminus of the building block and benzoyl instead of acetyl groups to protect the hydroxyls. The Pfp ester is stable under glycosylation reaction conditions but also reactive enough to be utilized directly in solid-phase synthesis [77]. Unfortunately, none of the above AFGP analogues has been tested for antifreeze activity.

Quite recently, Nishimura and his co-workers [73] carried out controlled polymerization at low temperature to synthesize cyclic AFGP analogues (cyAFGP) and observed the influence of molar mass, cyclic conformation, and the peptide *C*- and *N*-terminus on TH activity (Figure 5C). Interestingly, the activity data suggest that small cyclic AFGP induced higher TH values than do higher analogues, and that the *C*- and *N*-termini of the cyAFGPs do not play a key role in determining antifreeze activity [73]. Because routine production of AFGPs using conventional solid-phase peptide synthesis has been limited to small building blocks, microwave-assisted solid phase peptide synthesis was accessed by Heggemann *et al.* and Peltier *et al*. [74,75,78] in order to investigate the effect of the sequential mutation of peptide backbones on either RI or TH activity using a common monosaccharide building block (Figure 5D,E). These monosaccharide-based AFGP analogues with 3–4 repeating units and sequential variations of the primary structure using glycine, proline, and serine instead of alanine exhibit decreased antifreeze activity compare to the native AFGP sequence. These studies also suggest that the terminal galactose in native AFGP is not necessary for significant activity and underscore the importance of having periodic turns in the peptide sequence [74,75,78,79,80,81].

Recently, homodimeric native AFGP analogues were synthesized as pure oligomers through convergent disulfide bond formation to increase the density of the OH groups on AFGPs (Figure 5F). However, the loss of antifreeze activity of these homodimers suggests that the activity depends not only on the number of carbohydrate moieties but also on the intrinsic conformations of each analogue [76].

### 6.2. C-Linked AFGP Derivative

**Figure 6:**
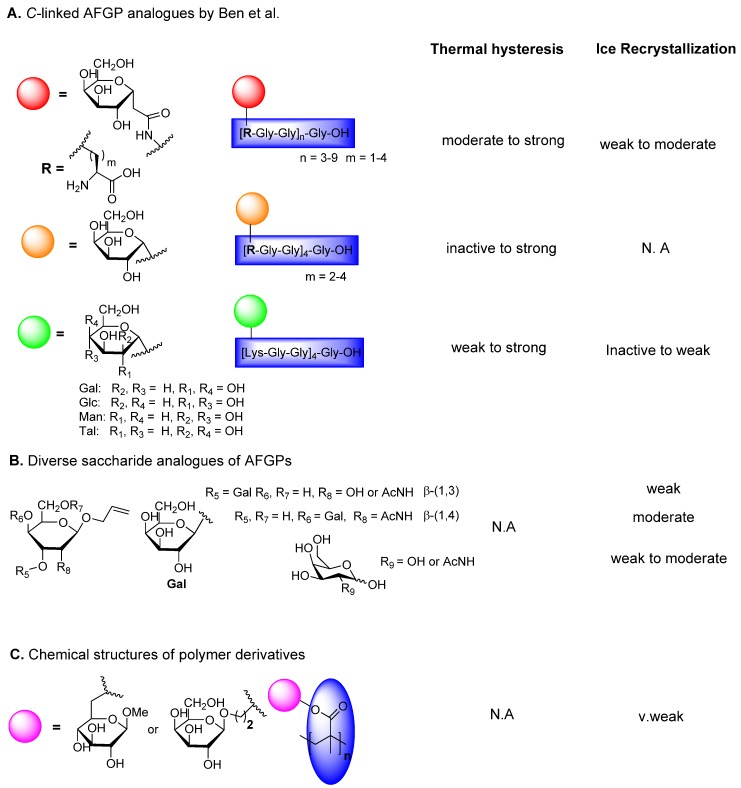
Synthesis of C-linked AFGP analogues and their antifreeze activities. Note: the figure is modified from [82,83,84,85,86,87,88,89,90,91].

Given the synthetic difficulties outlined above, Ben’s research group has focused on AFGPs analogues that are synthetically more accessible and contain stable *C*-glycosides in place of *O*-glycosides. Based on the presence of Arg in some of the natural AFGPs, a simplified monosaccharide in which the terminal galactose and the *N*-acetyl group from the native AFGPs were removed was attached to Lys as a *C*-linked glycoconjugate (Figure 6A). They systematically carried out the following SAR studies using 3 different series of C-AFGP analogues: (1) the effect of the peptide backbone structure, by replacing Ala and GalNAcThr residue using Gly and *C*-linked galactose attachment via an amide linker to Lys [82,83,84], (2) the effect of linker length between the backbone and the glycoside on activity, with different *C*-linker chain lengths either via an amide linker or utilizing the cross metathesis strategy [85,86,87], and (3) the effect of the type of the glycoside by varying the configuration of the carbohydrate moiety using glucose, mannose, and talose (Figure 6B) [88,89,90].

Although the earlier reported synthesis was linear, a more efficient method of convergent synthesis for *C*-linked AFGPs was reported by the same group in 2001 that included fewer steps, and the peptide backbone was glycosylated in the final step of the synthesis [82,83,84]. All the *C*-linked analogues with 3, 6, or 9 repeats of the tripeptide unit were tested for their TH, ice growth habit modification and RI properties. Of these compounds, a few of the *C*-linked AFGP analogues are potent inhibitors of recrystallization and do not possess TH activity (Figure 6A). Samples were assayed for RI activity using the “splat-cooling” method. Because SAR studies show that increasing the length of the linker and the substitution of galactose with glucose, mannose, and talose resulted in the complete loss of RI activity, the configuration of the carbohydrate and the length of the *C*-linked glycopeptide appears to be important for RI activity [82]. In addition, mechanistic studies on *C*-linked AFGPs suggest that carbohydrate hydration is important in RI activity and that RI activity is correlated to cryopreservation ability.

Further studies on the relationship between conformation, hydration, and RI activity in a series of amide-linked analogues suggest that changes in the backbone conformation and the spatial relationship between the carbohydrate moiety and the peptide backbone could play a key role in the activity of these molecules [86,88,89]. Another interesting feature of *C*-linked AFGPs is that one of the most potent *C*-linked analogues was rapidly internalized in cells and showed no or little *in vitro* cytotoxicity and apoptotic death inhibition [87]. The same group recently investigated the role of β-d-galactosyl-(1,3)-α-d-*N*-acetylgalactosamine in ice recrystallization activity by studying closely related structures, including those of β-linked (1,3)-, (1,4)-, and (1,6)-galactosyl-*N*-acetyl galactosamine and the β-(1,3)-galactosyl-galactoside (Figure 6B). The RI properties of these analogues were measured, and it was demonstrated that the β-linked-(1,4) disaccharide exhibits more potent RI activity than the native disaccharide. In contrast to the disaccharide analogues, the presence of the C2 *N*-acetyl group in monosaccharaides decreased the activity, suggesting that the group at C1 modulates RI activity [90]. Another novel design of carbohydrates that inhibit ice recrystallization has been carried out by Gibson *et al.* [91] using water-soluble polymers in order to determine the molar mass dependency on RI activity. However, these polymers, PMAMGlc and PGalEMA, were not as active as AFGP or the C-linked analogues, thus highlighting the fact that the density of OH groups on the sugar is less important that the distance of the OH groups from the backbone (Figure 6C) [91].

### 6.3. Triazole-Linked AFGP Derivatives

**Figure 7:**
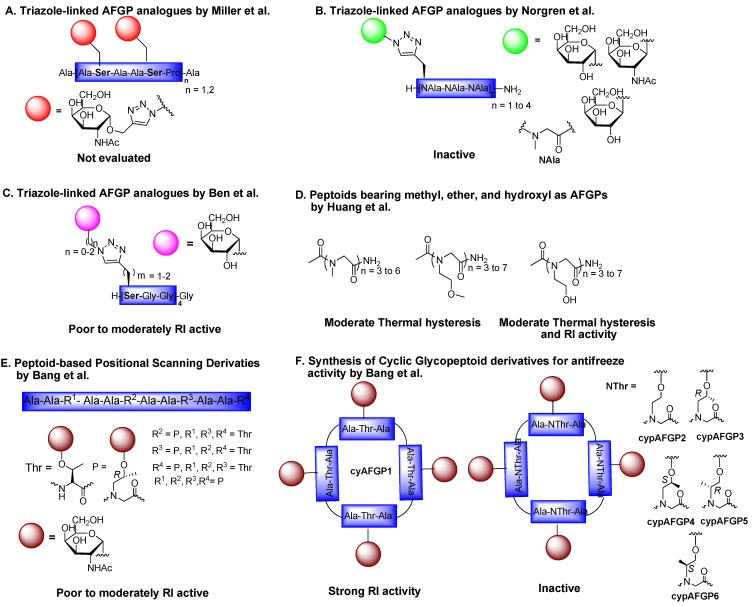
Synthesis of trizole and peptoid-derived AFGP analogues and their antifreeze activities. Note: the figure is modified from [92,93,94,95,96,97].

Another flexible platform reported by three different research groups for the synthesis of a wide range of antifreeze glycopeptide analogues used click chemistry as an alternative. In the first convergent synthesis reported by Miller *et al*. [92], triazole-linked AFGP analogues were assembled between α-propargylated d-*N*-acetyl galactose and azidoalanine using microwave radiation, and the corresponding glycopeptides were tested for activity. However, the effect of this triazole linker modification on TH or ice growth inhibition is unknown [92]. In the second approach, peptoid glycoconjugates with carbohydrate moieties attached by CuI-catalyzed azide alkyne cycloaddition (CuAAC) was undertaken by Norgen *et al* [93]. The analogues were prepared by building the peptoid backbone using Fmoc-protected alkyne-substituted peptoid monomers and then the glycosylation reaction was carried out using click chemistry. Similar to the first approach, the loss of activity of these peptoid glycoconjugates could be because of either the click linker or a difference in the conformation of the peptoid [93] (Figure 7B). More recently, Ben’s group also reported the generation of triazole-linked AFGP analogues in an attempt to mimic the amide bond in the C-AFGP analogue using the above convergent solid-phase synthesis approach. In contrast to the first 2 approaches, some of the triazole-containing AFGP analogues showed moderate RI activity, which suggests that a hydrophobic component (*i.e.*, the methylene spacer) close to the sugar residue, as well as the length of the side chain bearing the triazole linkage, plays key roles in the activity [94] (Figure 7C).

### 6.4. Peptoid Mimics

Bang’s research group has been focused on developing *N*-linked peptoid analogues of antifreeze glycoproteins, arguing that the glycosidic *O*-linkage attaching the sugar to the peptide backbone is a labile bond and the difference in the conformation of the peptoid may produce drastic changes in antifreeze activity [95,96]. This is the first study that shows the direct comparison of the antifreeze activities of *N*-linked α-d-*N*-acetylgalactosamine (α-d-GalNAc)-containing glycopeptoid derivatives. Peptoids can be described as mimics of α-peptides in that the side chain is attached to the backbone amide nitrogen instead of the α-carbon. Because of their ability to confer high conformational flexibility, high structural diversity, and improved proteolytic stability, peptoids have been developed as a new peptide modification strategy. In order to generate metabolically stable glycopeptide analogues without impairing antifreeze activity, we reported a highly efficient convergent strategy in which the glycosylated peptoid building blocks (*N*^α^-Fmoc-Ala-NThr (Ac3-α-d-GalNAc) or *N*^α^-Fmoc-Ala-NSer (Ac3-α-d-GalNAc)) were prepared using glycosylated Fmoc-protected peptoid monomer and the commercially available Fmoc-l-Ala-OH in the presence of DIC. The key glycosylation reaction was carried out using an Fmoc-protected peptoid monomer (Fmoc-NThr-OtBu) with 3,4,6-tri-*O*-acetyl-2-azido-α-d-galctopyranosyl bromide in the presence of Ag_2_CO_3_ [95]. Subsequently, the assembly of glyco-peptoid monomer into linear AFGP analogues was performed using standard Fmoc-based solid-phase peptide synthesis. Initially these analogues were subjected to positional scanning analysis in order to figure out the distinct position of peptoid mimics in native AFGP with either increased or retained activity. A few analogues showed moderate ice recrystallization activity, but the remaining AFGP peptoid mimics possessed no RI activity [95] (Figure 7D). Because cyclization is considered a useful strategy to gain stability and also decreased the flexibility of peptoid mimics, thereby eventually enhancing the bioavailability, cyclic antifreeze glycopeptide and glycopeptides through *N*-linked glyco-threonine were synthesized and tested for RI activity. In accordance with previous findings, native cyAFGP showed strong RI activity even though the disaccharide was replaced with a monosaccharide, whereas the peptoid derivatives (cypAFGPs) did not show any activity [96] (Figure 7E). More recently, using standard submonomer solid phase peptide synthesis, a small library of peptoids ranging from trimers to heptamers with side chains bearing hydroxyl (Ac(NSer)*n*), ether (Ac(Nme)*n*), and methyl (Ac(Sar)*n*) substituents were synthesized to compare the effects of backbone structure, side chain chemical composition, and sequence length on antifreeze activity (Figure 7F). Using a 2D X-ray microdiffraction method, the authors observed a reduction in melting temperature; thus, these peptoid analogues can display non-colligative antifreeze activity, most likely through specific binding to ice crystal growth interfaces. They concluded that the observed “dual-action” antifreeze activity in terms of ice growth inhibition and melting temperature reduction depends both on the oligomer backbone structure and on side-chain chemical composition [97].

## 7. Application Studies

AF(G)Ps can decrease the freezing temperature of solutions and inhibit ice recrystallization during freezing and thawing procedures [98]. These unique abilities have been tested for potential applications in biotechnology, cell biology, medicine, the food industry, *etc*. Moreover, AF(G)Ps are considered less toxic or nontoxic cryoprotective agent (CPA) candidates because they depress the freezing point non-colligatively, so that a much lower amount could be used than in the case of toxic chemical CPAs. In this section, the state of research into the development of AFP-based preservation or cryopreservation studies will be described.

### 7.1. Cryopreservation

In general, the cryopreservation of cell lines using general cryoprotectants is well established. The high amount of CPAs used in the cryopreservation of cells and tissues diminishes freezing damage, but also results in cytotoxicity [99]. Even though many studies have been carried out on the cryopreservation of a variety of cell lines, reproductive cells, tissues, and organs using AF(G)Ps, the results have not been revealed accordingly [40,100].

AF(G)Ps could play a role in the survival of organisms by preventing damage caused by recrystallization. Many attempts to develop promising hypothermic storage or cryopreservation techniques have been carried out on bovine and porcine oocytes [101,102], mouse oocytes, sperm, embryos [103], porcine embryos [104], *in vivo*-matured metaphase II oocytes [105], vertebrate and invertebrate cell lines [106,107], animal embryos [108], oyster (*Crassostrea gigas*) oocytes [109], carp sperm [110], ovine sperm [111], sheep embryos [112], bull sperm [113], seabream embryos [114], chimpanzee (Pan troglodytes) spermatozoa [115], zebrafish embryo, PGC cells [116], rice embryogenic suspension cells [117], human embryonic liver/kidney cells [87], islet cells [118], platelets [119], *E. coli* [120], and pig oocytes [121].

It has also been proposed that the cryopreservation property of AF(G)Ps may come from the interactions of proteins with the cell membrane [101,102,122,123,124], and another group suggested that cryopreservation was due to ion leakage inhibition by blocking potassium and calcium ion channels, as a result of the binding of AFPs and cell membranes of oocytes and embryos [101,102,121,122,123]. Further research has revealed that AFGPs prevent the leakage of dielaidoylphosphatidylcholine (DEPC), dielaidoylphosphatidylethanolamine (DEPE), and dielaidoylphosphatidylglycerol (DEPG) [53,124,125,126]. Moreover, it has been shown that AFGP7-8 protected spinach thylakoid membranes and dimyristoylphosphatidylcholine (DMPC) membranes by supplementation of galactolipids as these membranes are cooled through the phase transition temperature during chilling [125]. Contrasting results have also been observed, indicating that AFPs, including type I AFP- and AFGP1-5, induced membrane leakage in a concentration-dependent manner [125,127]. Negative cryopreservation effects of AF(G)Ps have also been demonstrated in plant cells [128,129], human red blood cells [130], ram spermatozoa [131], equine embryos [132], mouse embryos [133,134], livers [135], and rat hearts [136].

RBCs have a significant impact on both the military and civilian communities with respect to transfusion. Because RBCs were first stored using citrate and glucose in 1916 [137], other studies have been carried out to prolong the storage [138,139,140,141]. To date, the maximal storage period in a non-frozen condition is six weeks, so other preservation strategies such as cryopreservation are required. Frozen RBCs can be stored for up to decade, which means that cryopreservation can be a good alternative to prolong the RBC storage period. However, RBC cryopreservation presents several potential problems, including solution effects, extra/intracellular ice formation, and dehydration. AF(G)Ps can address the ice recrystallization issue. Studies have revealed the cryopreservation effect of AF(G)Ps on RBCs by minimizing ice recrystallization in the presence of HES or glycerol [142,143,144,145,146].

Organs can be stored hypothermically up to 72 h depending on the organ type, but they undergo gradual deterioration during cold storage [147,148]. Several studies have reported that the TH activity of AFPs minimized cold-induced injury [149,150]. Rat livers were preserved by AFGP perfusion before hypothermic storage [151]. Mammalian hearts were stored at subzero temperatures using type I and type III AFPs [151]. AFP treatment improved survival and hemodynamics, and reduced cell death, which is obviously better than the standard preservation procedure. However, some studies reported conflicting results, showing that the use of AFGP could be harmful in cardiomyocytes or heart preservation [136]. The reason was defined in a later study showing that needle-like ice, formed by AFGP, penetrated the cardiomyocytes and killed the cells during freezing [151].

Even though the problem of this negative effect of AFGP has not been solved, this issue could possibly be addressed by peptide or protein engineering through modifying the needle-like ice structuring property of AFGP. This technology might not be readily available before fully understanding the structure-function relationship of AFGP, but it is promising as results of synthetic analogues are being accumulated.

### 7.2. Cryosurgery & Food Preservation

Considering the fact that AFP-induced needle-like ice crystals destroyed the cells [143], AFPs have great beneficial effects for cryosurgery when used at higher concentrations. Type I AFP-driven intracellular ice crystals destroyed human primary prostatic adenocarcinoma cells [106,152], and subcutaneous tumors of mice were destroyed to a much better extent than in the case of treatment without type I AFP injection prior to freezing [153,154].

The ice RI activity of AFP has been successfully applied to food preservation. Studies have reported that AFPs are effective in preserve food texture through ice recrystallization inhibition during freezing and storage [38,155,156]. This recrystallization inhibition ability of AFPs has been utilized in dairy products such as ice cream and yogurt (Breyers from Unilever™, and Haagen-Dazs™ from Edy’s). It was also shown that animal muscle treated with AFP or AFGP prior to freezing reduced the resulting cryo damage [157]. Moreover, lamb muscle injected with AFGP before slaughtering revealed reduced drip loss of the meat [158]. In addition, the application of AFP to food preservation may lower the storage temperature and inhibit the growth of bacteria that may have contaminated the food prior to freezing [159].

### 7.3. Transgenic Approaches

The AFP gene may confer the property of cold tolerance to transgenic organisms. Previous studies have shown that transgenic salmon constructed by the introduction of type I AFP into eggs revealed freeze-resistant traits, and tissue-specific seasonal AFP expression was identified [160,161]. A transgenic goldfish was established by type III AFP microinjection, and this animal exhibited significant cold resistance [162]. Several studies established transgenic plants: Type I AFP was introduced into tomato [163], potato [164], wheat [165], and tobacco [163,166], and type II AFP was introduced into tobacco [167]. Moreover, type III AFP-transplanted transgenic mouse ovaries were successfully cryopreserved by vitrification [168,169].

## 8. Conclusions and Outlook

Despite over 30 years of studying antifreeze (glyco) proteins, there are still several issues such as cytotoxicity, low abundance in nature, and stability that must be overcome before AFGPs can find widespread application. First, a significant limitation of native AFGP is dynamic ice shaping/TH (assuming they are intrinsically related), which exacerbates cellular damage by altering crystals to form long specular ice crystals [106,170,171]. Second, in contrast to AFP types I and III, AFGP 8 is cytotoxic to human embryonic liver and kidney cells at concentrations higher than 2 and 0.63 mg/mL [172,173,174]. Finally, the maximum concentration to enhance the cryoprotective effect against red blood cells and islet cells using AFGPs is limited by the onset of increased cell damage due to specular ice crystal formation [118,143]. However, the negative effect of specular ice crystal growth was successfully used in cryosurgery using AFPs [175]. These results suggest that the cytotoxicity of AFGP8 is not only concentration-dependent but also cell specific. Thus, the structure of the AF(G)Ps, the freezing protocol, cell type, and reported cell viabilities of dimethyl sulfoxide vary dramatically between studies, making it difficult to ascertain the true ability of these compounds to protect cells. Towards this end, rationally designed *C*-linked Xaa-Gly-Gly mimics have delivered novel compounds that display interesting RI properties and low toxicities towards human embryonic liver and kidney cells but do not suppress the freezing temperature or inhibit nucleation in the same way that biological antifreeze compounds do. In contrast to the activity of triazole- and *N*-linked peptoid mimics, these compounds are also stable against hydrolysis and amenable to the scale up production of limited number of amino acid coupling using solid phase synthesis. Further structural modifications on these mimics in terms of optimizing flexibility could modulate their antifreeze properties. A recent study on the stabilization of membranes during the cooling of phospholipid membranes using AF(G)Ps indicates that higher molecular weight AFGPs confer increased protection to the cell membranes [126]. In this regard, the native chemical ligation technique used to make native AFGPs could be merged to synthesize the above synthetic mimetics to afford full-length AFGP analogues of unlimited size. AF(G)P mimics are already being used in frozen foods to prevent cellular destruction and improve the texture. These proteins appear to be non-allergenic and non-toxic but for any new material, the magnitude of its individual antifreeze property and its concentration need to be considered for translating into industrial application. Finally, the author believes that further evaluation of AFGP analogues in terms of additional functional group tolerance, and the effect of cryopreservation on a wide-range of cell lines, tissues, or organ samples will definitely accelerate the smooth transition from academic to industrial applications.
